# Colour vision in ADHD: Part 1 - Testing the retinal dopaminergic hypothesis

**DOI:** 10.1186/1744-9081-10-38

**Published:** 2014-10-24

**Authors:** Soyeon Kim, Mohamed Al-Haj, Samantha Chen, Stuart Fuller, Umesh Jain, Marisa Carrasco, Rosemary Tannock

**Affiliations:** Department of Applied Psychology & Human Development, OISE, University of Toronto, 252 Bloor Street West, Toronto, ON M5S 1 V6 Canada; Department of Psychology, York University, Toronto, Canada; Department of Clinical Psychology, University of Western Ontario, London, Canada; Department of Psychology, New York University, New York, USA; Department of Psychiatry, Centre for Addiction and Mental Health, Toronto, Canada; Department of Psychology and Neural Science, New York University, New York, USA

**Keywords:** ADHD, Color saturation, Contrast sensitivity, Sex difference

## Abstract

**Objectives:**

To test the retinal dopaminergic hypothesis, which posits deficient blue color perception in ADHD, resulting from hypofunctioning CNS and retinal dopamine, to which blue cones are exquisitely sensitive. Also, purported sex differences in red color perception were explored.

**Methods:**

30 young adults diagnosed with ADHD and 30 healthy young adults, matched on age and gender, performed a psychophysical task to measure blue and red color saturation and contrast discrimination ability. Visual function measures, such as the Visual Activities Questionnaire (VAQ) and Farnsworth-Munsell 100 hue test (FMT), were also administered.

**Results:**

Females with ADHD were less accurate in discriminating blue and red color saturation relative to controls but did not differ in contrast sensitivity. Female control participants were better at discriminating red saturation than males, but no sex difference was present within the ADHD group.

**Conclusion:**

Poorer discrimination of red as well as blue color saturation in the female ADHD group may be partly attributable to a hypo-dopaminergic state in the retina, given that color perception (blue-yellow and red-green) is based on input from S-cones (short wavelength cone system) early in the visual pathway. The origin of female superiority in red perception may be rooted in sex-specific functional specialization in hunter-gather societies. The absence of this sexual dimorphism for red colour perception in ADHD females warrants further investigation.

**Electronic supplementary material:**

The online version of this article (doi:10.1186/1744-9081-10-38) contains supplementary material, which is available to authorized users.

## Introduction

Attention-Deficit Hyperactivity Disorder (ADHD) is the most frequently diagnosed psychiatric disorder in childhood, with worldwide prevalence rates estimated at 5.3% [1]. Longitudinal studies show that approximately 65% of children with ADHD continue to show symptoms in adulthood [1–3]. Surprisingly, despite the high prevalence and detrimental impact of ADHD, its underlying pathophysiology remains unclear.

Current theories posit that executive function deficits account for many of the poor outcomes in ADHD, which are supported by evidence of delayed maturation and functional anomalies in the prefrontal-striatal circuitry that underpin executive functioning. However, accumulating evidence attests to anomalies in other cortical circuits in ADHD, including the visual network, suggesting that executive dysfunction may not be the dominant neurobiological characteristic of ADHD e.g., [4].

Behavioural manifestations of visual perceptual problems, particularly color perception problems have been associated with ADHD [5–8]. Color perception problems in ADHD, particularly problems with the color blue, have been explained in terms of the ‘retinal dopamine hypothesis’, which posits that a deficiency in central nervous system dopamine induces a hypo-dopaminergic state in the retina, which in turn would have deleterious effects on short wave-length cones (‘blue’; S-cones) that are scare in number and very sensitive to dopamine, as well as other neurochemical agents [9]. Collectively, the preceding findings indicate the need for further investigation of visual function and its regulation by attentional processes in ADHD. In this paper we report data pertaining to color perception in young adults with ADHD; in our companion paper, we report the effects of attention on color perception in these individuals.

Previous studies of color perception in ADHD, including our own [8], have focused on hue discrimination in ADHD. Hue refers to the specific tone of a color (i.e. red, blue, green). However, hue is only one of the three characteristics used to describe color. Another key characteristic is saturation, which refers to the intensity or purity of a given hue. A pure monochromatic light is fully saturated; adding white light dilutes it and decreases saturation. In this study, we sought to expand the scope of previous findings on hue discrimination ability in adults with and without ADHD by also examining color saturation discrimination ability. We also investigated whether there are sex differences in color saturation and contrast sensitivity within ADHD (female ADHD vs. male ADHD) and control groups (female control vs. male control), as well as between the groups (female ADHD vs. female control, male ADHD vs. male control).

From an evolutionary perspective, it has been proposed that females who served as the primary gatherers may have developed superior red color perception that would allow them to better distinguish among fruits, foliage and insects [10–12]. Moreover, if picking ripe fruits triggered the development of better color perception through genetic modulation, females may have retained remnants of superior red color discrimination abilities [13, 14]. However, studies of sex differences in color perception have yielded inconsistent results [12, 15–19]. One possible explanation is that most of these studies focused on hue discrimination, rather than color saturation, which is more likely to be important for foraging efficiency when searching for ripe fruits and edible leaves [20]. We hypothesized that control females would show superiority in red color saturation discrimination, which would reflect remnants of an evolutionary sex-specific functional behavior. Furthermore, we hypothesized that this sexual dimorphism would not be manifest in ADHD, given that observed alterations in sex-specific brain maturation patterns in ADHD may affect color perception [21–23]. Specifically, delayed pruning of dopamine receptors in female individuals with ADHD may cause a hypodopimanergic state which may affect color processing.

To measure color saturation and contrast sensitivity, we employed a paradigm which measures the ability to discriminate test stimuli that differ in saturation/contrast level [24, 25]. In this paradigm, two stimuli (a standard stimulus that has a constant saturation/contrast level and a test stimulus that varies in the level of color saturation/contrast sensitivity) are preceded by a cue that appears adjacent to the test, neutral, or standard stimulus location. Participants are asked to report the orientation of the grating of higher saturation/contrast (left or right). The critical manipulation is that observers are not asked to rate their subjective experience of the stimulus saturation/contrast, but to make a decision about the stimulus orientation. A cue in either the neutral (central) or peripheral location precedes the stimuli. Peripheral cue conditions are designed to manipulate exogenous attention, whereas the neutral cue condition does not direct attention to the stimuli locations and serves as a baseline to evaluate the effects of attention. To investigate color saturation/contrast sensitivity discrimination ability in ADHD we report data taken solely from the neutral cue condition; the companion paper reports data from the peripheral cue conditions.

**Figure 1:**
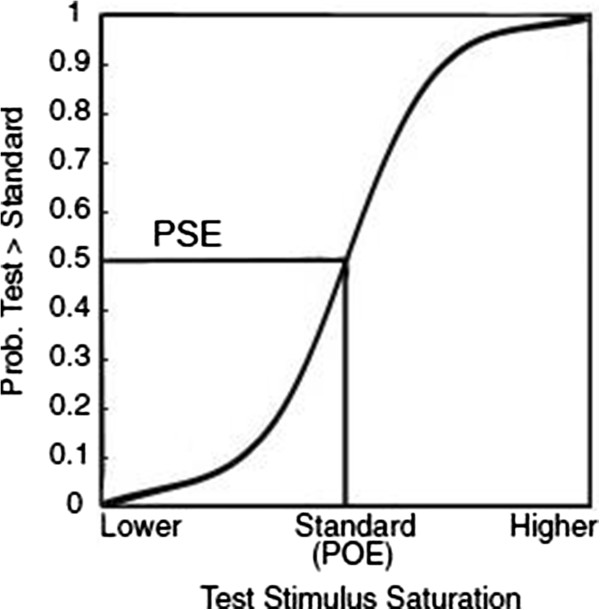
**PSE/POE.** The PSE is the “point of subjective equality”, when the two stimuli (Test and Standard) look subjectively the same, and thus, an observer would choose randomly between them. As such, the PSE is the 0.5 probability point. The POE refers to “point of objective equality” which corresponds to the ‘Standard’ stimulus saturation. The POE values are the median of the 11 saturation levels on the x-axis of the PSE graph. If exogenous covert attention (cue) has an effect on saturation perception, the PSE value would be significantly apart from POE. We expected that exogenous covert attention would enhance the saturation perception, hence, the test cue line would move towards the left, while standard cue line moves towards the right side. This change would indicate that due to the cue presented at the same side as the test stimuli, lower saturation level of the actual test stimuli would appear to have the same saturation level as the neutral stimuli.

Specifically, we examined three key variables: accuracy (percent of total responses that were correct in the neutral cue condition collapsed across saturation manipulation), slope (accuracy as a function of increasing saturation, see Additional file 1) and the difference between POE (“point of objective equality” which is the saturation/contrast value of the standard stimuli) and PSE (“point of subjective equality”, saturation/contrast level of the test stimuli at .5 probability; see Figure 1). The PSE in the neutral cue condition is expected to be approximately equal to the standard saturation/contrast value (POE).

We maintained constant luminance across the tasks, as well as between stimuli and background, and modified color stimuli to be ‘pure’. In addition, we excluded participants who reported color vision problems as well as those who had a family history of color deficiency to avoid possible genetic confounds stemming from an inclusion of heterozygous females [19, 26–28].

## Methods

### Participants

Thirty participants with ADHD (50% male; age range: 18–35 years old; mean age: 24 years old) were recruited through college and university accessibility services across the Greater Toronto Area. Inclusion criteria were: 1) current enrolment in a post-secondary program, 2) a previous diagnosis of ADHD, 3) registration with a respective university or college Student Disability Services, which requires documented evidence of a previously confirmed diagnosis of ADHD, 4) aged 19–35 and 5) right handedness. Exclusion criteria were: 1) uncorrected sensory impairment, 2) major neurological dysfunction and psychosis, 3) current use of sedative or mood altering medication, and 4) any known genetic or current vision problems present in first degree family members. Those who are treated with stimulant medication were required to stop any stimulant medication for at least 48 hours prior to the study. Thirty control participants matched on age and gender were recruited through advertisements posted at the same local university and community. All participants provided informed written consent before starting the study. The study was approved by the Institutional Research Ethics Board.

### Descriptive measures

All participants (i.e., those in the ADHD and control groups) were asked to complete the following measures to assess psychological/emotional distress, cognitive impairments and activation level that may affect task performance.

#### Adult ADHD Self-Report Scale (ASRS v1.1)

The ASRS [29] was administered to assess current ADHD symptoms. The ASRS is an instrument consisting of eighteen questions based on the criteria used for diagnosing ADHD in the DSM-IV-TR. Scores for each item are added to calculate a total score. The ASRS is a reliable and valid scale for evaluating ADHD in adults [30]. It has high internal consistency (Cronbach’s alpha 0.88 0.89, for both patient and rater-administered versions respectively) and high concurrent validity with the rater-administered ADHD Rating Scale. Informant reports (usually provided by a family member) were collected to confirm current symptoms of ADHD.

To ascertain the robustness of the students’ self-reported current symptoms in the ADHD group, we compared their ASRS total score with the ASRS total score reported by their designated significant other. Four significant others were not able to provide the ASRS survey for the participants due to personal issues such as travelling and language problems. There was no difference between the self-reported and significant-other scores [*F* (1, 24) = 1.531, *p* = .228, *ES* = .06]. Also, we compared participant scores on the 6-item ASRS Set A that was administered orally as part of the initial telephone intake interview (in which participants were required to provide examples of behaviour for each of the 6 items) with their scores for the first 6 items (Set A) from the standard questionnaire version of the ASRS: two of the participants did not complete Set A. Self-reported scores did not differ as a function of modality of gathering the information [*F* (1, 26) = 1.13, *p* = .297, *ES* = .042]. Note that about 30% of the ADHD sample in this study also contributed data to a larger-scale reliability study of the ASRS [31].

#### Kessler psychological distress scale (K10)

The K-10 [32] was used to examine recent emotional distress. The K10 is a 10-item questionnaire based on questions about anxiety and depressive symptoms that a person has experienced in the most recent 4-week period. Scores for each item were added to calculate a global measure of distress.

#### The Cognitive Failures Questionnaire (CFQ)

The CFQ measures self-reported failures in perception, memory, and motor function in everyday life. This 25-item measure has good external validity [33, 34]. Questions require subjects to rank how often these mistakes occur on a 5-point Likert scale. A total score was used to evaluate participant’s general cognitive functioning.

### The Thayer Activation-Deactivation Check List (AD-ACL)

The AD-ACL is a self-report measure for tonic alertness. The AD-ACL uses a visual analog scale (VAS) presented on paper [35, 36], which has been reported to have adequate reliability and validity [36, 37]. For each of Thayer’s adjectives, an 82-mm line is presented. Participants indicate their current feelings at that moment on the 82 mm bipolar VAS by making a slash mark perpendicular through the VAS line. The location of the slash mark (from “definitely feel” to “definitely do not feel”) was later measured in millimeters and assigned a score from 1 to 82.

### Visual function measures

#### Visual Activities Questionnaire (VAQ)

The VAQ was used to assess perceived visual function in ordinary activities [38]. The VAQ is a self-report questionnaire consisting of 33 items that are behaviorally based, in that they refer to actual visual activities and tasks. These 33 items fall into eight areas that are known to be important in carrying out visual activities: Color discrimination, Glare disability, Light/Dark adaptation, Acuity/Spatial vision, Depth perception, Peripheral vision, Visual search, and Visual processing speed. The VAQ total score was used to measure general visual activity difficulties. The VAQ is a reliable and valid scale for clinically evaluating visual difficulties [38, 39].

#### Farnsworth-Munsell 100 Hue Test (FMT)

The FMT was used to provide an objective assessment of hue discrimination ability [40]. The FMT is a widely used color vision test that requires participants to sequence color reference caps in order of incremental hue variation spanning the visible spectrum. All color caps are equally bright and their separation in color appearance is equidistant. The error scores reflect the number of misplacements. Error scores were computed separately for the blue, red, and green spectra. All testing was performed under standard light conditions in the same room and at the same place. The luminance was maintained at D65 daylight, 6500 degrees Kelvin.

### Color saturation/contrast discrimination task

This discrimination task was adapted from that used by Carrasco and colleagues [24] and Fuller and Carrasco [25] to measure discrimination ability and the role of exogenous covert attention in contrast sensitivity and color saturation, respectively. A central fixation point appeared on screen for 500 ms, followed by a cue (neutral or peripheral) for 67 ms. In this paper, only data for the neutral cue condition are reported: these data allow us to compare the ability to discriminate higher color saturation and contrast sensitivity in the ADHD and comparison group. Participants’ responses on the neutral cue condition measure their ability to discriminate color saturation and contrast discrimination under distributed attention conditions (i.e., in the absence of covert exogenous attentional shifts to the stimuli location). After a delay of 53 ms, two stimuli that differed in either color saturation or contrast were presented for 40 ms. Participants were required to determine whether the stimulus with the higher contrast (or saturation) was tilted to the left or right and to indicate their decision by pressing the appropriate response key. Note that with one key press participants convey information about both the stimulus orientation and its perceived contrast/saturation.

Modifications were made to the original paradigm to provide a more rigorous measurement of participants’ ability to discriminate the color saturation of blue and red stimuli by ensuring that all the other features of the task and stimuli remained the same. These modifications included: 1) stimuli were created to be exactly co-linear with the white points for their respective backgrounds (all backgrounds had the same chromatic and luminance coordinates) and more equally spaced in color-space; 2) ‘pure’ blue and red colored stimuli were created to better isolate the S – (L + M) and L-M opponent cone systems; 3) luminance (similar to ‘brightness’) was kept constant between tasks, as well as between stimuli and background; and 4) cue size and shape was the same across tasks (round dot, .40 degrees).

### Procedures

**Figure 2:**
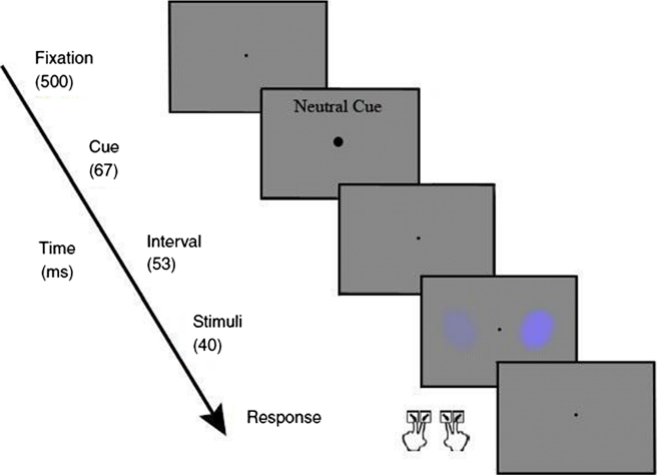
**Task design.** A central fixation (500 ms) was followed by a cue (neutral or peripheral): part one reports only data derived from the neutral cue-condition. After a delay of 53 ms, stimuli were presented for 40 ms. The short period of stimuli presentation preclude saccadic eye movement, allowing to access the influence of exogenous covert attention. In each trial, participants were instructed to answer the question, “Is the stimulus that looks higher in contrast tilted to the right or left?” or “Is the stimulus that looks more colorful tilted to the right or left?” Participants chose from four options and responded by pressing one of the four designated response keys: left stimulus, tilted to left (‘z’ key); left stimulus tilted to right (‘x’ key); right stimulus, tilted to left (‘n’ key); or right stimulus, tilted to right (‘m’ key).

The task schematic is shown in Figure 2. In each trial, participants were instructed to answer the question, “Is the stimulus that looks higher in contrast tilted to the right or left?” or “Is the stimulus that looks more colourful tilted to the right or left?” Participants responded by pressing one of the four designated response keys: left stimulus, tilted to left (‘z’ key); left stimulus, tilted to right (‘x’ key); right stimulus, tilted to left (‘n’ key); or right stimulus, tilted to right (‘m’ key). After ensuring that participants understood the instructions, participants were asked to complete 80 practice trials before completing the actual task (10 blocks). Practice trials were tested individually and feedback response accuracy (defined as selecting the correct response key for higher color saturation and orientation, or higher contrast and orientation) was shown at the end of the practice trials. The feedback on accuracy was only given in the practice session to ensure that the participants understood the task. Cut off scores of 80% in discrimination accuracy and in tilt accuracy had to be met before participants continued with the actual experimental tasks.

Participants were invited for two sessions which were conducted on two different days to avoid fatigue effect. The average interval between the sessions was 6 days, and no significant difference was found between the groups [Control: 6.35 ± 4.71, ADHD: 6.86 ± 3.88, *p* = .648]. In each session, participants completed a total of 10 blocks of trials (1056 trials in total; each block took approx. 3 minutes to complete) for each stimulus condition (blue, red, and contrast) yielding a total of 1056 trials (2 sessions = 2112 trials). Participants were encouraged to take a short break after finishing each block. Between each 10 blocks of a stimulus type (blue, red or contrast), the clinical descriptive (ASRS, K-10, CFQ, AD-ACL) and vision measures (FMT, VAQ) were administered in random order. The cue conditions (i.e. test, neutral, standard) were presented to participants in random order. Each cue condition consisted of a total of 704 trials over both days. Present analysis was conducted based on the 704 trials of the neutral cue condition.

### Stimuli

**Figure 3:**
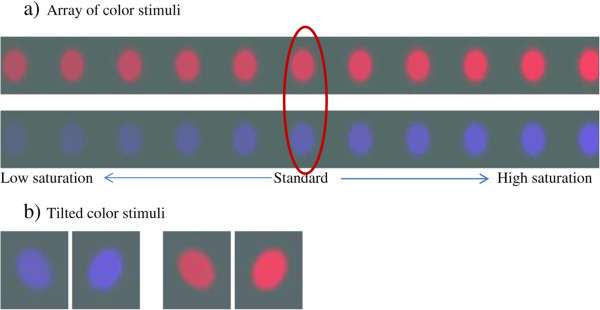
**Color stimuli.** Color stimuli varied in saturation level **(a)** and were subtended 2^○^ of visual angle to either left or right side **(b)**. Luminance was kept equal for both red and blue stimuli and background. Color stimuli were modulated by a clipped Gaussian envelope to blend the edges with the background and minimize border effects.

Color stimuli consisted of a uniformly-colored (blue or red) patch modulated by a modified Gaussian envelope that was elongated vertically and clipped at half-height (see Figure 3). This served to blend the edges of the stimuli chromatically with the background, while retaining uniform color saturation in the middle. Two types of stimuli were presented randomly on each side (left and right). One stimulus was designated as the standard while the other was the test stimulus. Standard blue stimuli had a fixed color saturation (DKL saturation 1.40) whereas the saturation level of the blue test stimuli varied among .50, .68, .86, 1.04, 1.22, 1.40, 1.58, 1.76, 1.94, 2.12, 2.30 (11 levels). Likewise, standard red stimuli had a fixed color saturation (DKL saturation 0.35) and red test stimuli varied among 0.25, 0.27, 0.29, 0.31, 0.33, 0.35, 0.37, 0.39, 0.41, 0.43, 0.45 (11 levels). A total of 2112 trials were collected for each task which allowed 192 trial points per each saturation level.

For contrast stimuli, a standard Gabor (a sinusoidal grating enveloped in a Gaussian window) was presented at 3 cycles per degree (cpd) spatial frequency (approximately .36 of the total size in degrees). Standard stimuli had a fixed contrast level (28.2%), whereas the contrast level of the test stimuli varied among 10%, 12.3%, 15.14%, 18.62%, 22.91%, 28.18%, 34.67%, 42.66%, 52.48%, 64.57%, 79.43% (11 levels).

For all color and contrast tasks, the side of the monitor that the standard stimuli were presented on (right or left visual field; RVF, LVF) and the level of the test stimuli were randomly distributed. This random presentation allowed us to further explore any visual field advantage (i.e., hemisphere advantage) for processing blue or red color saturation, as well as contrast sensitivity. The size of each stimulus was .36 (horizontal) × .57 (vertical) × 3° and located at 4° eccentricity. They were tilted 20° either to left or right. The fixation point was a 0.15° black dot. The neutral cue was a 0.4° black dot (100 cd/ m2) located in the center of the display. For more detailed description of stimuli and task, see Fuller & Carrasco [25] and the Additional file 2.

### Apparatus

The stimuli, which were generated using Matlab (MathWorks, Natick, MA) and custom code, were displayed on a 21-in. Dell LCD monitor (1024 ” 768 pixels at 75 Hz) with Asus 64- bit operating system. The monitor was calibrated using a Photo research PR650 SpectraColorimeter (Chatsworth, CA) and Matlab calibration routines from the PsychToolbox3.

### Analysis

Data points with *SD*’s greater than 3 were regarded as outliers and adjusted using a winsorizing technique [41]. Two data points on VAQ questionnaire and FMT were winsorized. Only the data on neutral condition (without attentional manipulation) were used to calculate accuracy. In the discrimination task data, psychometric function covering less than .70 and tilt accuracy less than .80 were excluded (two participants from each group). Although the mean tilt accuracy of all three stimuli were above .90 in both groups, the control group was more accurate in tilt discrimination than the ADHD group with the blue stimuli [*F* (1, 56) = 10.93, *p* = .002] and the red stimuli [*F* (1, 56) = 5.98, *p* = .016], but no difference was found with the contrast stimuli.

A one-way ANOVA on descriptive measures such as ASRS, K-10, CFQ and AD-ACL between the groups was performed. Vision measures such as VAQ total score and FMT (blue, red, green and yellow) were also compared through one-way ANOVAs between the groups.

Color saturation discrimination task data were analyzed in three steps to test for differences among groups, sexes, and hemispheres (note that the present analyses only used data from the neutral cue condition):

In Step 1, we first assessed the test-retest reliability of the color saturation/contrast discrimination task, using a 3 (cue type: test, neutral, standard) × 2 (session: first, second) × 2 (group: ADHD, control) repeated measures ANOVA. Main effect of cue type was present in colour saturation and in contrast [blue: *F* (2, 57) = 23.860, *p* = .000; red: *F* (2, 57) = 3.199, *p* = .048, contrast: *F* (2, 57) = 15.209, *p* = .000], which will be discussed in the Part 2 manuscript in more details. Most of the interactions involving Cue type were not significant; Cue type by session [blue: *F* (2, 57) = 1.268, *p* = .289; red: *F* (2, 57) = .287, *p* = .752; contrast: *F* (2, 57) = 2.574, *p* = .085], or Cue type by Group [red: *F* (2, 57) = 1.016, *p* = .368; contrast: *F* (2, 57) = 1.174, *p* = .316; except for blue [*F* (2, 57) = 3.614, *p* = .033]. Importantly, there was no main effect of session [blue: *F* (1, 58) = .165, *p* = .686; red: *F* (1, 58) = .813, *p* = .372; contrast: *F* (1, 58) = .032, *p* = .859], and none of the interactions between session and group were significant [blue: *F* (1, 58) = .001, *p* = .976; red: *F* (1, 58) = .640, *p* = .427; contrast: *F* (1, 58) = 1.158, *p* = .286]. We believe that the reason we did not find a main effect of session in our paradigm is because the paradigm tests basic perceptual processing that require very fast stimuli presentation. Thus, in this paper, as in previous studies using this paradigm [42–44] we report the data for the neutral condition combined across the two sessions.

Step 2 tested for group differences as well as sex differences between the ADHD and control groups on 3 key variables: accuracy, defined as the percent of total responses that were correct; slope, which measures accuracy as a function of increasing saturation, and POE-PSE data (point of subjective equality - point of objective equality which are supposed to be the same in the neutral cue condition). Specifically, separate 2 (Group: control, ADHD) × 2 (Sex: male, female) × 3 (Stimuli: red, blue, contrast) repeated measures ANOVAs were conducted for accuracy, slope, and POE-PSE to determine the effect of group, sex and stimuli in the neutral cue condition.

In Step 3 we tested for possible visual field/hemispheric advantages in color saturation discrimination or contrast sensitivity. In this analysis, we used accuracy data for each visual field (left vs. right). Specifically, we used% of correct response when the test stimuli was presented in each side. We conducted three separate 2 (Hemisphere: right, left) × 2 (Group: ADHD, control) repeated measures ANOVAs with accuracy data for each stimulus to explore possible visual field/hemispheric advantages in color saturation discrimination and contrast sensitivity.

Cohen’s *d* was reported to measure the standardized magnitude of group differences, which is relatively insensitive to sample size. Conventionally, Cohen’s *d* ranging from 0.2 to 0.3 is considered to be a small effect size, and a Cohen’s *d* of 0.5 and 0.8 are considered to index medium and large effect sizes, respectively.

## Results

### Descriptive measures

As expected, ADHD participants reported significantly more symptoms on the ASRS than the comparison group [*F* (1, 58) = 77.50, *p* < .001], as well as more problems in everyday cognitive functioning as measured using the CFQ [*F* (1, 57) = 47.46, *p* < .001] and higher emotional distress on the K10 [*F* (1, 57) = 10.21, *p* = .002]. The two groups did not differ in levels of alertness or tonic arousal as evaluated with the AD-ACL (see Table 1).

**Table 1:** **Summary data for participant characteristics and clinical measures of color vision**

Measures	Controls (n = 30)		ADHD (n = 30)		ANOVA		Cohen’s ***d***
	***M***	***SD***	***M***	***SD***	***F***	***p***	
ASRS	23.63	7.55	47.00	12.42	77.50	.00	2.27
K10	28.60	13.23	36.93	4.76	10.21	.002	.84
GRIT	53.47	11.21	61.00	7.23	9.56	.003	.80
CFQ – Total score	31.07	10.84	54.67	15.05	47.46	.00	1.80
AD- ACL	337.00	87.48	310.65	89.31	1.33	.25	.30
VAQ - Total score	14.35	3.29	15.21	3.90	.84	.37	.24
FMT							
Overall time	473.48	95.93	536.14	136.79	4.08	.048	.53
Red (Error)	7.82	7.32	5.88	5.59	1.30	.26	.30
Blue (Error)	9.05	7.40	11.29	6.79	1.47	.23	.32
Green (Error)	15.73	9.50	16.66	8.11	.16	.69	.11
Yellow (Error)	7.97	6.33	6.53	6.48	.74	.39	.22

### Vision and color perception in ADHD

One-way ANOVAs conducted on the VAQ total score revealed no significant difference between the groups (as can be seen from the summary data shown in Table 1). Hue discrimination ability was analyzed with one-way ANOVAs for each color spectrum (red, green, blue, and yellow) on the FMT error scores. Individuals with ADHD did not differ from the control participants in the number of errors on any color spectrum of the FMT. Analysis of the time to complete the FMT revealed slower color discrimination in the ADHD group than in the comparison group [*F* (1, 56) = 4.08, *p* = .048].

### Perception of color saturation/contrast sensitivity in ADHD

#### Color saturation/contrast discrimination

A 2 × 2 × 3 repeated measures ANOVA in accuracy revealed a significant main effect for Group [*F* (1, 52) = 5.61, *p* = .022, *ES* = .097] and Stimuli [*F* (2, 51) = 21.18, *p* < .001, *ES* = .45]. The main effect of Sex was not significant, but there was a significant Sex × Group interaction [*F* (1, 52) = 5.01, *p* = .030, *ES* = .088]. None of the other interactions were significant: Group × Stimuli interaction [*F* (2, 51) = .829, *p = .442*, *ES* = .031], Sex × Stimuli interaction [*F* (2, 51) = 1.494, *p* = .234, *ES* = .055], and Group × Sex Stimuli × Stimuli interaction [*F* (2, 51) = .005, *p* = .995, *ES* = .000].

To explore the significant Sex × Group interaction and also to test our a-priori hypotheses concerning group differences in color perception accuracy within male and female groups (i.e., poorer blue saturation discrimination in ADHD group), we conducted separate one-way ANOVAs for each stimuli within each sex and also within groups. *Within females*, these analyses revealed that ADHD participants were less accurate in color discrimination for blue saturation [*F* (1, 28) = 6.30, *p* = .018] and red saturation [*F* (1, 28) = 14.01, *p* = .001] than control participants, but no sex differences were present in contrast sensitivity accuracy. Analysis of the *slope* parameters is consistent with the findings from the analysis of accuracy. Specifically, female control participants showed a steeper slope for blue [*F* (1, 28) = 5.41, *p* = .027] and red [*F* (1, 28) = 7.55, *p* = .010] saturation in comparison to female ADHD participants. This difference was not found for the contrast discrimination task. *Within males,* no significant difference in discrimination accuracy was found between male participants in the ADHD and control groups across the three stimuli (blue, red and contrast). No significant difference in slope was found among the male participants in the ADHD and control groups across the three stimuli (blue, red and contrast). Results of the *POE-PSE* analysis revealed no significant sex difference between male and female participants, indicating no sex-related perceptual biases.

**Figure 4:**
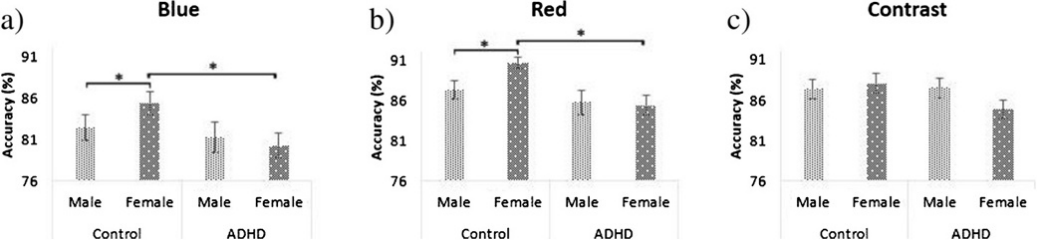
**Accuracy bar graph between sexes.** Percentages of correct responses for each stimulus (**a**-Blue, **b**-Red, and **c**- Contrast) are compared between the groups in separate lines for male and female. Females in control groups show superiority in blue and red color saturation discrimination while no difference was shown in contrast sensitivity or in females with ADHD.

Next, we tested for the a-priori hypothesis that female controls would show superior red color saturation discrimination compared to males, by conducting one-way ANOVAs on three stimuli separately within the control group and within the ADHD group. *Within the control group,* accuracy data in the neutral cue condition revealed that female participants were significantly more accurate in discriminating higher red saturation than males [*p* = .02; *ES* = -.95]. No sex differences were found for blue saturation discrimination or for contrast discrimination (see Table 2, Figure 4). Similarly, one-way ANOVA analyses using *slope* data showed significant difference in red [*p* = .03; *ES* = -.84; the slope was steeper for females than males]. No sex differences were found for blue color saturation discrimination or contrast discrimination in slope. Lastly, there was no significant difference between the sexes for the magnitude of difference between POE and PSE in the neutral cue condition for each stimulus (blue, red, and contrast). Analysis *within the ADHD group* revealed no significant sex differences in terms of accuracy, slope and POE-PSE for each stimulus (blue, red, and contrast) in the neutral cue condition (all *p* > .1).

**Table 2:** **Means and standard deviations of accuracy, POE-PSE and slope in male and female participants**

	Control (***n*** = 29)		ADHD (***n*** = 29)	
	***M***	***SD***	***M***	***SD***
Accuracy				
Blue				
Male	82.53	5.99	81.36	6.63
Female^a^	85.44	5.03	80.30	6.04
Red				
Male	87.34	4.35	85.81	5.32
Female^a,b^	90.73	2.57	85.45	4.69
Contrast				
Male	87.35	4.57	87.51	4.38
Female	88.09	4.51	84.92	4.35
POE-PSE				
Blue				
Male	.062	.049	.082	.044
Female	.088	.047	.084	.076
Red				
Male	.004	.003	.006	.004
Female	.004	.004	.005	.003
Contrast				
Male	.059	.020	.069	.018
Female	.065	.016	.072	.033
Slope				
Blue				
Male	3.37	1.63	3.20	1.82
Female^a,b^	3.90	1.40	2.82	1.13
Red				
Male	9.25	2.49	8.69	3.98
Female^a^	11.44	2.70	8.65	2.84
Contrast				
Male	-3.23	1.04	-3.44	.93
Female	-3.46	.83	-2.86	1.07

^a^Control Females > ADHD Females, ^b^Control Females > Control Males.

#### Hemispheric differences

There were no main effects of group, hemisphere, or Hemisphere × Group interaction in blue discrimination accuracy. A main effect of group was present [*F* (1, 56) = 8.22, *p* = .01, *ES* = .13] in red (control > ADHD), whereas no main effect of hemisphere or Hemisphere × Group interaction was present. Lastly, there was a significant main effect of hemisphere for contrast discrimination [*F* (1, 55) = 23.38, *p* < .00, ES = .30]. A post hoc analysis indicated that accuracy was significantly higher when contrast stimuli were presented to the LVF/RH. Neither the main effect of group nor the Group × Hemisphere interaction was significant (see Table 3).

**Table 3:** **Means and standard deviations of hemispheric accuracy**

	Control		ADHD	
	M	SD	M	SD
Blue (*n* = 29/29)				
Right hemisphere	84.38	6.49	81.33	6.32
Left hemisphere	83.49	5.75	80.60	6.29
Red (*n* = 29/29)				
Right hemisphere	89.12	4.66	85.52	5.86
Left hemisphere	88.83	4.03	85.04	5.86
Contrast (*n* = 29/28)				
Right hemisphere	88.52	4.02	87.50	4.06
Left hemisphere	87.01	5.46	84.75	5.49

## Discussion

The present study yielded two major findings: 1) only females with ADHD showed impairments in both blue and red color saturation discrimination compared to controls, but they did not differ in contrast sensitivity (Table 2, Figure 4); and 2) a sexual dimorphism for red color perception was found only in the control group, in which females showed superior perception of red color saturation compared to males: no such sexual dimorphism was observed in the ADHD group (Table 2, Figure 4). Also, no differences were found in terms of hue discrimination between individuals with ADHD and control participants, although the ADHD group took longer overall to complete the FMT. Analysis of hemispheric differences indicated that all participants showed higher accuracy on contrast discrimination for stimuli presented to the LVF/RH.

### Retinal dopaminergic hypothesis

Our findings are only partly consistent with the retinal dopaminergic hypothesis of color vision, which posits that the ADHD group should be less accurate in distinguishing blue saturation compared to controls, but would not differ in either red or contrast discrimination abilities [9]. By contrast, our results indicate that color perception anomalies were restricted to females with ADHD. Specifically, they were not only less accurate in blue saturation discrimination compared to female controls but also in red saturation discrimination, although no differences were found in contrast sensitivity. We begin by presenting a theoretical framework (i.e. the multistage color vision model) that would help account for perceived differences in blue and red saturation discrimination abilities. Then we shall examine why color perception differences occurred in females with ADHD but not in males with ADHD.

The seemingly contradictory finding of differences in both red and blue color perception in females with ADHD can be explained by the multistage color vision model [45, 46], which in essence, views the formation of the color systems as occurring at a later cortical stage at which L/M and S/LM opponent processes are combined. This was further clarified and expanded upon by Mancuso et al. [47] by explaining the cellular mechanism of this process based on evolutionary and retinal anatomical perspectives. Taking into account that M-cones are a new addition to the existing S-cone system, the new M-cones might have been incorporated into a pre-existing blue-yellow circuit to create red-green color vision. Specifically, red perception would be derived from a neural comparison between (S + L)-M; green from M-(S + L); blue from (S + M)-L and yellow from L-(S + M). Based on this multi-stage color vision theory, S-cone not only influences perception of blue color, but also other colors such as green, yellow and red [45–48]. This S-cone input early in the visual pathway may play a vital role in correcting the apparent imbalance between L-M cone (consisting 90-95% of the cone population) and S-cone color systems (5-10% of the cone population; Mancuso et al. [47]). In sum, because color perception of all four colors (blue, yellow, red, and green) is based on input from S-cones early in the visual pathway, we may speculate that the reduced accuracy seen in ADHD on red saturation perception could have resulted from the same effect of a postulated hypodopaminergic effect on S-cones.

### Sexual dimorphism in color saturation discrimination

Only female ADHD participants showed significantly poorer accuracy in colour saturation discrimination compared to control female participants; no significant differences were present between male ADHD and control participants. Also, a phenotypic sex difference in discriminating red color saturation was found within control group, which was not present in the ADHD group.

A sexual dimorphism in red color perception found in the control group can be explained with an evolutionary perspective which proposes that sex-specific functional behaviour in human hunter-gatherer societies would have allowed females to better distinguish ripe fruit [10, 49, 50]. Specifically, the extensive experience of searching for highly saturated red (ripe) fruits as a gatherer may have contributed to the development of superior red saturation discrimination ability in females. For example, saturation has been suggested to be more effective in identifying a ripe fruit than hue [20]. Also, the fact that L-M cone photopigment coding genes are located on the X chromosome may have been advantageous for L-M opponent cone processes in females [12, 51]. Furthermore, the emotional aspect of gathering ripe fruits could have played a role in the development of more accurate discrimination ability. Ecological valence theory [52] explains that color preference may be derived from adaptive functions to survive and positive emotions may have developed as a result of an adaptive behaviour. Perhaps the delicious savour and fulfilling emotions that may have resulted from picking ripe fruit further enhanced accuracy in red saturation discrimination among females.

Notably, females with ADHD did not manifest this advantage in red saturation discrimination and showed poorer discrimination accuracy in both red and blue compared to females in control group. The absence of this sexual dimorphism in ADHD females may be attributable to a higher density and delayed pruning of dopamine receptors in the striatum found in females with ADHD during peri-adolescence [23]. Extensive dopamine reuptake process due to overproduction of dopamine synapses and receptors in the central nervous system (CNS) is considered to be one of the reasons for ADHD symptoms [53]. Andersen and Teicher [23] observed that the overproduction of D1 and D2 family receptors rapidly normalizes in peri-adolescent years with extensive pruning in males but not in females (rodent). Human data using magnetic resonance imaging (MRI) also shows that the stratum shrinks in adolescence for males but not for females [54]. Likewise, female dopamine receptor density declined at a slower rate than males in adulthood [55]. This delayed pruning of DA receptors in female may preclude the opportunity to reduce overproduction of DA receptors in female young adults with ADHD. Given that dopamine has an important role in chromatic perception [56, 57], we speculate that delayed pruning of presynaptic dopamine re-uptake receptors in females with ADHD would result in a hypo-dopaminergic state, which may contribute to poorer color perception compared to female peers.

### Hue discrimination

Although group differences in color saturation discrimination were significant, no reliable differences were found for hue discrimination as measured by the FMT. Our results are inconsistent with findings of previous studies which reported hue discrimination differences for individuals with ADHD, specifically in the ability to discriminate blue-yellow stimuli [5–8]. One possible explanation for the discrepant findings is that previous studies did not control for color vision problems in first-degree family members. The possible inclusion of females who are carriers of a color vision defect (heterozygotes) would have confounded the results by affecting chromatic sensitivity [26, 27]. Furthermore, the FMT has been considered to be a semi-quantitative evaluation test with various limitations [58], such as decreased sensitivity and poor reliability, compared to psychophysical techniques that allow for very fine adjustments of the stimulus parameters. Not only did Farnsworth [40] note that a 30% change in scores was possible from one test session to another, but this poor test-retest reliability was also demonstrated by Birch and colleagues [59]. Moreover, the FMT requires the participant to arrange the caps in the best color order (i.e. from yellowish green to turquoise green). This process involves both accurate movement execution and sustained attention, which are suggested to be impaired in ADHD. In sum, more sensitive tests that allow fine contrast adjustments should be used to clarify the issue of hue discrimination in the ADHD population.

Our study also revealed the both the ADHD and control groups showed subtle, but significantly higher accuracy on contrast discrimination for those stimuli presented to the LVF/RH. Our results on RH superiority in contrast discrimination are in line with findings from several previous studies [60–64]. Recently, Okubo and Nicholls [60] suggested that the RH has more flexible contrast gain control mechanisms than the LH which allows it to process spatial frequency effectively over a wide range of contrast levels. In sum, our findings support the notion that early evoked activities in the RH are relatively more sensitive to spatial frequency than the LH.

The present study has some limitations. First, we did not verify the presence or absence of familial vision problems, nor could we verify the absence of heterozygosity in X-linked cone photopigment expression or its nature (protan vs. deutan) in female participants, which could influence chromatic sensitivity. Second, our findings may not generalize to the larger populations of adults with ADHD. Given that our participants were recruited from post-secondary institutions, they may have greater functioning than those who have not received post-secondary education, and thus, may not share the characteristics of the general population of adults with ADHD. Third, we did not confirm either the ADHD diagnosis, nor did we assess for comorbid disorders (e.g., specific learning disorders), which may themselves be associated with color perception problems e.g., [65]. Lastly, we provided feedback in the practice trials to ensure participants understood the instruction, but it may have served to calibrate their responses to the feedback. Replication and further research is required to confirm the observed color perception anomalies in ADHD and to specify underlying mechanisms.

Keeping in mind the aforementioned limitations, the current study provided evidence of decreased perceptual abilities in females with ADHD in terms of discriminating the saturation of pure red and pure blue colors, but no differences in perception of contrast in achromatic stimuli. Our findings suggest that the retinal dopaminergic hypothesis requires modification: namely to account for the fact that human perception of red implicates input from both L and S cone receptors. Also, our finding suggests that the color perception anomalies are restricted to female ADHD. Furthermore, given that attention is known to influence visual perception e.g., [24, 25], it is possible that the observed perceptual difficulties in ADHD may be attributable, at least in part, to impairments in selective attention – a question we address in our companion paper.

## Electronic supplementary material

Additional file 1: **Glossary.** (DOCX 54 KB)

Additional file 2: **DKL coordinates for blue and red stimuli.** (DOCX 17 KB)
