# Supplementary material for: Colour vision in ADHD: Part 1 - Testing the retinal dopaminergic hypothesis
Source: Behav Brain Funct. 2014 Oct 24;10:38. doi: 10.1186/1744-9081-10-38 (PMC4219036; doi:10.1186/1744-9081-10-38)
Supplement: Supplementary file 1 — Additional file 1: Glossary. (DOCX 54 KB) [file 12993_2014_505_MOESM1_ESM.docx]

**Additional file 1 Glossary**

**Slope:** The function used for the fitting is a cumulative Weibull function.  The specific equation used in this study to calculate slope variable is as following:

y = γ + (1 - γ - λ).*(1 - exp(-1*(x./ α).^ β))

Gamma (γ) is the lower bound, lambda (λ) is the error or lapse rate (these simply allow for the asymptotes not to be 0 and 1).  The x values are the stimulus values, alpha (α) is a scaling parameter and beta is the slope parameter, and the dependent variable here y is the proportion data for each of the stimulus values.  The beta (β) exponent controls the rate of rise or fall in y with the change in x.

**DKL color space**: A widely used color space, “Derrington-Krauskopf-Lennie” (DKL) space, in which the coordinates represent the purported responses of the three second-site colour discrimination mechanism, L+M, L–M, and S–(L+M). The modulation directions that change the response of one of these mechanisms while leaving the response of the other two fixed are referred to as “cardinal directions.”


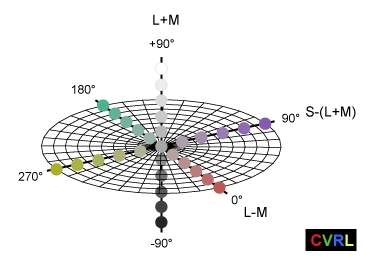


**Supplement Figure 1. DKL color space.** The grid corresponds to the isoluminant plane, which includes the L-M (0º - 180º) and S (90º - 270º) cardinal axes. The vertical axis is the achromatic L+M+S axis (-90º - +90º). The representations of the colors along each axis are approximate, but notice that the unique hues do not align with the cardinal axes. [Source of the DKL figure: Stockman, A., & Brainard, D. H. (2009). Color vision mechanisms. In M. Bass, C. DeCusatis, J. Enoch, V. Lakshminarayanan, G. Li, C. Macdonald, V. Mahajan & E. van Stryland (Eds.), The Optical Society of America Handbook of Optics, 3rd edition, Volume III: Vision and Vision Optics. New York: McGraw Hill.]
