# Supplementary material for: Colour vision in ADHD: Part 1 - Testing the retinal dopaminergic hypothesis
Source: Behav Brain Funct. 2014 Oct 24;10:38. doi: 10.1186/1744-9081-10-38 (PMC4219036; doi:10.1186/1744-9081-10-38)
Supplement: Supplementary file 2 — Additional file 2: DKL coordinates for blue and red stimuli. (DOCX 17 KB) [file 12993_2014_505_MOESM2_ESM.docx]

**Additional file 2. DKL coordinates for blue and red stimuli**

| **Table 1.1. Blue x,y,Y coordinates** | | | |  | **Table 1.2. Blue spherical DKL** | | | |  | **Table 1.3. Blue DKL values (azimuth- the hue)** | | | |
| --- | --- | --- | --- | --- | --- | --- | --- | --- | --- | --- | --- | --- | --- |
| **Saturation level** | **x** | **y** | **Y** |  | **Saturation level** | **x** | **y** | **Y** |  | **Saturation level** | **Achromatic** | **LM** | **S** |
| 1 | 0.2713 | 0.2739 | 30 |  | 1 | 0 | 1.5708 | 0.5000 |  | 1 | 0 | 0 | 0.5000 |
| 2 | 0.2653 | 0.2569 | 30 |  | 2 | -0.0000 | 1.5708 | 0.6800 |  | 2 | -0.0000 | -0.0000 | 0.6800 |
| 3 | 0.2600 | 0.2419 | 30 |  | 3 | -0.0000 | 1.5708 | 0.8600 |  | 3 | -0.0000 | -0.0000 | 0.8600 |
| 4 | 0.2553 | 0.2286 | 30 |  | 4 | -0.0000 | 1.5708 | 1.0400 |  | 4 | -0.0000 | -0.0000 | 1.0400 |
| 5 | 0.2511 | 0.2166 | 30 |  | 5 | 0 | 1.5708 | 1.2200 |  | 5 | 0 | 0 | 1.2200 |
| 6 | 0.2473 | 0.2058 | 30 |  | 6 | 0.0000 | 1.5708 | 1.4000 |  | 6 | 0.0000 | 0.0000 | 1.4000 |
| 7 | 0.2439 | 0.1960 | 30 |  | 7 | 0.0000 | 1.5708 | 1.5800 |  | 7 | 0.0000 | 0.0000 | 1.5800 |
| 8 | 0.2408 | 0.1872 | 30 |  | 8 | 0.0000 | 1.5708 | 1.7600 |  | 8 | 0.0000 | 0.0000 | 1.7600 |
| 9 | 0.2379 | 0.1791 | 30 |  | 9 | 0.0000 | 1.5708 | 1.9400 |  | 9 | 0.0000 | 0.0000 | 1.9400 |
| 10 | 0.2353 | 0.1717 | 30 |  | 10 | -0.0000 | 1.5708 | 2.1200 |  | 10 | -0.0000 | -0.0000 | 2.1200 |
| 11 | 0.2329 | 0.16487 | 30 |  | 11 | -0.0000 | 1.5708 | 2.3000 |  | 11 | -0.0000 | -0.0000 | 2.3000 |
| x,y: chromatic coordinates, Y: Luminance in candela | | | |  | x,y: chromatic coordinates, Y: Luminance in candela | | | |  | LM: Long-Medium wavelength cones (i.e. red); S: Short wavelength cones (i.e. blue) | | | |

| **Table 2.1. Red xyY coordinates** | | | |  | **Table 2.2. Red spherical DKL** | | | |  | **Table 2.3. Red DKL values (azimuth- the hue)** | | | |
| --- | --- | --- | --- | --- | --- | --- | --- | --- | --- | --- | --- | --- | --- |
| **Saturation level** | **x** | **y** | **Y** |  | **Saturation level** | **x** | **y** | **Y** |  | **Saturation level** | **Achromatic** | **LM** | **S** |
| 1 | 0.3894 | 0.2847 | 29.0247 |  | 1 | 0 | 0 | 0.25 |  | 1 | 0 | 0.25 | 0 |
| 2 | 0.39604 | 0.2812 | 28.9466 |  | 2 | 0 | 0 | 0.27 |  | 2 | 0 | 0.27 | 0 |
| 3 | 0.40264 | 0.2778 | 28.8686 |  | 3 | 0 | 0 | 0.29 |  | 3 | 0 | 0.29 | 0 |
| 4 | 0.4091 | 0.2743 | 28.7906 |  | 4 | 0 | 0 | 0.31 |  | 4 | 0 | 0.31 | 0 |
| 5 | 0.4154 | 0.2710 | 28.7126 |  | 5 | 0 | 0 | 0.33 |  | 5 | 0 | 0.33 | 0 |
| 6 | 0.4216 | 0.2678 | 28.6346 |  | 6 | 0 | 0 | 0.35 |  | 6 | 0 | 0.35 | 0 |
| 7 | 0.4277 | 0.2646 | 28.5565 |  | 7 | 0 | 0 | 0.37 |  | 7 | 0 | 0.37 | 0 |
| 8 | 0.4336 | 0.2614 | 28.4785 |  | 8 | 0 | 0 | 0.39 |  | 8 | 0 | 0.39 | 0 |
| 9 | 0.4395 | 0.2583 | 28.4004 |  | 9 | 0 | 0 | 0.41 |  | 9 | 0 | 0.41 | 0 |
| 10 | 0.4453 | 0.2553 | 28.3224 |  | 10 | 0 | 0 | 0.43 |  | 10 | 0 | 0.43 | 0 |
| 11 | 0.4509 | 0.2523 | 28.2444 |  | 11 | 0 | 0 | 0.45 |  | 11 | 0 | 0.45 | 0 |
| x,y: chromatic coordinates, Y: Luminance in candela | | | |  | x,y: chromatic coordinates, Y: Luminance in candela | | | |  | LM: Long-Medium wavelength cones (i.e. red); S: Short wavelength cones (i.e. blue) | | | |
